# Optimizing the Intracellular Delivery of Therapeutic Anti-inflammatory TNF-α siRNA to Activated Macrophages Using Lipidoid-Polymer Hybrid Nanoparticles

**DOI:** 10.3389/fbioe.2020.601155

**Published:** 2021-01-14

**Authors:** Abhijeet Lokras, Aneesh Thakur, Abishek Wadhwa, Kaushik Thanki, Henrik Franzyk, Camilla Foged

**Affiliations:** ^1^Department of Pharmacy, Faculty of Health and Medical Sciences, University of Copenhagen, Copenhagen, Denmark; ^2^Department of Drug Design and Pharmacology, Faculty of Health and Medical Sciences, University of Copenhagen, Copenhagen, Denmark

**Keywords:** lipidoid-polymer hybrid nanoparticles, RNAi - RNA interference, siRNA - small interfering RNA, quality-by-design, TNF-α, macrophage, drug delivery, nanomedicine

## Abstract

RNA interference (RNAi) has an unprecedented potential as a therapeutic strategy for reversibly silencing the expression of any gene. Therapeutic delivery of the RNAi mediator, i.e., small interfering RNA (siRNA), can be used to address diseases characterized by gene overexpression, for example inflammatory conditions like chronic obstructive pulmonary disease (COPD). Macrophages play a key role in COPD pathogenesis and are recruited to the airways and lung parenchyma, where they release proinflammatory cytokines, e.g., tumor necrosis factor-alpha (TNF-α). Hence, targeting TNF-α with siRNA is a promising therapeutic approach for COPD management. However, a safe and effective delivery system is required for delivery of TNF-α siRNA into the cytosol of hard-to-transfect macrophages. The purpose of this study was to optimize the intracellular delivery of TNF-α siRNA to the lipopolysaccharide-activated murine macrophage cell line RAW 264.7 using lipidoid-polymer hybrid nanoparticles (LPNs) composed of the lipid-like transfection agent lipidoid 5 (L_5_) and the biodegradable polymer poly (D,L-lactide-co-glycolide). Applying a quality-by-design approach, the influence of critical formulation variables, i.e., the L_5_ content and the L_5_:siRNA ratio (w/w), on critical quality attributes (CQAs) was investigated systematically using risk assessment and design of experiments, followed by delineation of an optimal operating space (OOS). The CQAs were identified based on the quality target product profile and included size, polydispersity index, zeta potential, encapsulation efficiency and loading for achieving efficient and safe TNF-α gene silencing in activated RAW 264.7 cells. Formulations inducing efficient gene silencing and low cytotoxicity were identified, and the optimal formulations displayed L_5_ contents of 15 and 20% (w/w), respectively, and an L_5_:siRNA weight ratio of 15:1. All tested formulations within the OOS mediated efficient and sequence-specific TNF-α gene silencing in RAW 264.7 cells at TNF-α-siRNA concentrations, which were significantly lower than the concentrations required of non-encapsulated TNF-α-siRNA, highlighting the benefit of the delivery system. The results also demonstrate that increasing the loading of siRNA into the delivery system does not necessarily imply enhanced gene silencing. This opens new avenues for further exploitation of LPNs as a robust platform technology for delivering TNF-α siRNA to macrophages, e.g., in the management of COPD.

## Introduction

RNA interference (RNAi) is a regulatory pathway in eukaryotic cells in which gene expression is inhibited at the messenger RNA (mRNA) level by sequence-specific double-stranded RNA, for example small interfering RNA (siRNA) (Ryther et al., 2005). In principle, any disease characterized by protein overexpression may be treated using synthetic molecules harnessing the RNAi pathway by Watson-Crick base-pairing, e.g., siRNA directed against a specific mRNA. Although the ability of double-stranded RNA to mediate silencing of gene expression was discovered in *Caenorhabditis elegans* more than two decades ago (Elbashir et al., 2001), only four drugs based on siRNA have been approved to date, i.e., patisiran (Adams et al., 2018), givosiran (Balwani et al., 2020), lumasiran (McGregor et al., 2020), and inclisiran (Raal et al., 2020; Ray et al., 2020) used for systemic treatment of the polyneuropathy of hereditary transthyretin amyloidosis, acute hepatic porphyria, and elevated LDL cholesterol respectively. Major hurdles for unlocking of the full potential of siRNA for therapeutic applications are delivery-related challenges (Dammes and Peer, 2020). These include, but are not limited to, (i) protection of siRNA against degradation by exo- and endonucleases, (ii) cellular uptake, and (iii) endosomal escape and siRNA release in the cytosol after cellular internalization (Haussecker, 2014). Some of these challenges have been partially overcome by chemical modification of the siRNA, which has enhanced the resistance to nucleases (Place et al., 2012). However, while chemical modification has certainly improved the drug properties of siRNA, the adoption of delivery technologies has also appeared to be essential for overcoming barriers related to siRNA delivery (Whitehead et al., 2009).

One example of a disease that can be targeted via the RNAi pathway is chronic obstructive pulmonary disease (COPD). More than 210 million people are affected by COPD, and by 2030, COPD is expected to be the fourth largest cause of death worldwide (Bousquet and Kaltaev, 2007). COPD severely compromises breathing, which causes a general decline in organ function, eventually resulting in chronic co-morbid conditions, including cardiovascular diseases, diabetes, and hypertension (Mannino et al., 2008). The pathophysiology of COPD is relatively complex and ranges from cellular inflammation to structural remodeling (Chung and Adcock, 2008). Tumor necrosis factor-alpha (TNF-α), along with interleukin 1-beta (IL-1β), plays a critical role in the inflammatory cascades during exacerbations of COPD (Matera et al., 2010; Cazzola et al., 2012). However, two clinical trials indicated no immediate or long-term benefit for patients with moderate to severe COPD of systemic treatment with the anti-TNF-α monoclonal antibody Infliximab (Rennard et al., 2007, 2013). Also, there was no significant difference between subjects treated with the corticosteroid prednisone compared to the TNF inhibitor Etanercept (Aaron et al., 2013). Hence, there is an urgent need to design novel treatment approaches for COPD, mainly focusing on the underlying inflammatory and irreversibly destructive phases. Targeting alveolar macrophages through RNAi mediated by siRNA represents one strategy to knock down the expression of inflammatory genes in COPD, e.g., TNF-α (Barnes, 2003; Peer and Lieberman, 2011). Therefore, we have previously exploited the RNAi machinery inherent in macrophages to ameliorate the gene expression of TNF-α with subsequent reduction of lipopolysaccharide (LPS)-induced inflammation (Jansen et al., 2019).

Overall, two types of delivery technologies are commonly used to deliver siRNA across the cell membrane into the cytosol (Roberts et al., 2020): i.e., (i) delivery systems like lipoplexes (Schroeder et al., 2010), lipid nanoparticles (LNPs) (Xu and Wang, 2015), cyclodextrin polymeric nanoparticles (Dominique et al., 2016), and lipid-polymer hybrid nanoparticles (LPNs) (Hadinoto et al., 2013), and (ii) conjugates (Kanasty et al., 2013), e.g., N-acetyl-D-galactosamine (GalNAc)-siRNA conjugates, which are used for liver targeting in the clinic (givosiran patisiran, lumasiran, and inclisiran) by actively targeting the internalizing asialoglycoprotein receptor 1 expressed by hepatocytes (Balwani et al., 2020). In general, LNPs represent the most clinically advanced delivery system for nucleic acids (Cullis and Hope, 2017), and patisiran, the siRNA in Onpattro, is delivered to liver hepatocytes using an LNP formulation (Adams et al., 2018). Lipid-based delivery systems are biocompatible and display high loading and transfection efficiency, but a drawback is their premature drug leakage and poor colloidal stability. Lipids with single or multiple cationic centers have been used as carriers for siRNA (Schroeder et al., 2010; Zhi et al., 2013). LNPs containing a single amine-based cationic lipid, a polyethylene glycol-lipid, and helper lipids, were first reported for intracellular delivery of siRNA (Heyes et al., 2005). Lipid-like materials referred to as lipidoids have also been developed for siRNA delivery, and they contain one or several amine centers and multiple hydrophobic tails (Akinc et al., 2009). Lipidoids consist of an alkylated tetraamine backbone, and depending on the degree of alkylation, different subtypes are obtained, e.g., the penta-substituted lipidoid, which is referred to as L_5_ (Akinc et al., 2009). To improve the efficiency of siRNA delivery, amino alcohol-based lipidoids have been developed (Love et al., 2010). On the other hand, delivery systems based on polymeric matrix systems, e.g., poly(lactic-co-glycolic acid) (PLGA) nanoparticles, display higher structural integrity and collidal stability, and enables sustained release of siRNA, but they display poor loading capacity and low transfection efficiency (Cun et al., 2010, 2011).

Therefore, we have designed LPNs for delivering siRNA by combining lipidoids with PLGA (Thanki et al., 2017) to exploit the advantages of both lipids and polymers. Hence, siRNA-loaded lipidoid-modified PLGA nanoparticle are biocompatible, have high loading and transfection efficiency, high colloidal stability, and allow for sustained siRNA release (Thanki et al., 2019a). In these LPNs, the anionic siRNA is complexed with the outer lipidoid shell, and also with the PLGA core by incorporating some of the net-neutral lipophilic complexes of siRNA and lipidoid (Colombo et al., 2015). Recent mechanistic uptake studies suggest that LPNs are taken up *via* macropinocytosis (Jansen et al., 2019) and probably because of leaky macropinosomes (Meier et al., 2002), improve lipidoid-mediated siRNA delivery and the resulting gene silencing (Love et al., 2010). However, bulk lipidoids are potent agonists for toll-like receptor (TLR) 4 and activate murine antigen-presenting cells (APCs) *in vitro (de Groot et al.*, *2018**)*. The agonistic effect was also confirmed *in silico*. Interestingly, this agonistic effect was abrogated when L_5_ was formulated as LPNs loaded with siRNA. However, no systematic studies have been performed to date to investigate the effect of the L_5_ content and the L_5_:TNF-α siRNA weight ratio on the *in vitro* safety and gene silencing effect of LPNs. Hence, we used a systematic quality-by-design (QbD) approach implementing risk assessment and design of experiments (DoE) for optimizing the loading of therapeutically relevant TNF-α siRNA in LPNs. The QbD approach and DoE are valuable tools as maximal information is provided from the least number of experiments. The QbD process involves the following steps: (i) identification of the quality target product profile (QTPP) i.e., the product attributes that are critical to stability, safety and efficacy; (ii) identification of the critical quality attributes (CQA); (iii) identification of critical process parameters (CPP), and (iv) layout and implementation of DoE to establish the relationship between the CQAs and the CPPs. This information is used to define a process design space i.e., the optimal operating space (OOS) that will result in an end product of the desired QTPP (Ingvarsson et al., 2013; Colombo et al., 2018). We also evaluated the safety and gene silencing mediated by TNF-α siRNA-loaded LPNs *in vitro* in the murine macrophage cell line RAW 264.7 and show that loading a higher dose of siRNA in the LPNs does not correlate with higher gene silencing *in vitro*.

## Materials and Methods

### Materials

2′-*O*-Methyl-modified dicer substrate asymmetric siRNA duplexes directed against TNF-α siRNA (17928.334 g/mol) and negative control siRNA were generously provided by GlaxoSmithKline (Stevenage, UK) as dried, purified and desalted duplexes (Supplementary Table 1). The siRNA duplexes were re-annealed according to a standard protocol recommended by Integrated DNA Technologies (IDT) (Coralville, IA, USA). PLGA (lactide:glycolide molar ratio 75:25, M_w_: 20 kDa) was obtained from Wako Pure Chemical Industries (Osaka, Japan). L_5_ was synthesized, purified, and characterized as previously reported (Akinc et al., 2008). Polyvinylalcohol (PVA) 403 with an average molecular weight of 30–70 kDa (87–90% degree of hydrolysis) was purchased from Sigma-Aldrich (St. Louis, MO, USA). Heparin and octyl β-D-glucopyranoside (OG) were obtained from Biochrom GmbH (Berlin, Germany) and Sigma-Aldrich, respectively. Quant-iT™ RiboGreen® RNA Reagent and Tris–EDTA buffer (TE buffer, 10 mM Tris, 1 mM EDTA, pH 8.0) were acquired from Molecular Probes, Invitrogen (Paisley, UK). Primers (Supplementary Table 1) were obtained from TAG Copenhagen (Copenhagen, Denmark). RNase-free diethyl pyrocarbonate (DEPC)-treated Milli-Q water was used for all buffers and dilutions. Additional chemicals were of analytical grade and purchased from Sigma-Aldrich and Merck (Copenhagen, Denmark).

### Preparation and Physicochemical Characterization of LPNs

L_5_-modified LPNs loaded with TNF-α siRNA were prepared using the double emulsion solvent evaporation (DESE) method, essentially as reported previously (Thanki et al., 2017, 2019b; Dormenval et al., 2019). The L_5_ content relative to the total solid content (L_5_ + PLGA) was varied from 15 to 25% (w/w), and the L_5_:TNF-α siRNA ratio ranged from 5.0:1 to 15.0:1 (w/w). The final batch size per formulation was 15 mg. The physicochemical properties (*z*-average, PDI, zeta potential, encapsulation efficiency, and siRNA loading) of the LPNs were determined as described previously (Thanki et al., 2017, 2019b; Dormenval et al., 2019).

### Statistical Optimization of LPNs

Initially, the effect of L_5_:TNF-α siRNA ratio was investigated on the physicochemical properties by keeping the L_5_ content constant and vice versa. Based on these experiments, 12 and 23-run response surfaces were constructed for TNF-α gene silencing, i.e., the half-maximal inhibitory concentration (IC_50_) values and physicochemical properties, respectively, using an I-optimal design with two critical independent variables, i.e., L_5_ content and L_5_:TNF-α siRNA weight ratio (Supplementary Table 2). The L_5_ content ranged from 15 to 25% (w/w), while the latter ranged from 5.0:1 to 15.0:1 (Table 1). Seven responses [*z*-average, PDI, zeta potential, encapsulation efficiency, siRNA loading, and the effective concentration for half- maximal gene silencing (IC_50_)] to the independent variables were measured. Critical responses were identified and subjected to model fitting by using analysis of variance (ANOVA), and the best model fit was selected based on statistical parameters, i.e., the *p*-value, the *R*^2^, the difference between adjusted and predicted *R*^2^, and the adequate precision. The results were further optimized numerically and graphically to construct desirability and overlay plots, respectively. Statistical optimization was performed using Design Expert (version 12.0.1, Stat-Ease Inc., Minneapolis, MN, USA).

**Table 1:** Formulation design space.

**Factor**	**Low level**	**Center level**	**Center-high level**	**High level**
L_5_ content (%, w/w LPNs)	15	20	-	25
L_5_:TNF-α siRNA weight ratio	5.0:1	7.5:1	10.0:1	15.0:1

### *In vitro* Gene Silencing

The murine macrophage cell line RAW 264.7 was purchased from the American Type Culture Collection (TIP71, Manassas, VA, USA). The cells were maintained in Dulbecco's Modified Eagle's Medium with high (4.5 g/L) glucose (DMEM+, Fisher Scientific Biotech Line, Slangerup, Denmark) supplemented with 100 U/mL penicillin, 100 μg/mL streptomycin, 2 mM glutamine (all from Sigma-Aldrich), and 10% (v/v) fetal bovine serum (FBS, Gibco, Life Technologies). The cells were grown in a 5% CO_2_-95% atmospheric air incubator at 37°C, the growth medium was renewed every second day, and the cells were subcultured twice a week by detaching them from the culture flask (75 cm^2^, Sigma Aldrich) using a cell scraper. Cells were seeded in 6-well tissue culture plates (Sigma Aldrich) at a density of 1.0 × 10^6^ cells/well. Subsequently, nanoparticle suspensions were added to each well resulting in final siRNA concentrations of 2.8, 5.6, 11.3, 27.9, and 55.8 nM, respectively, in duplicates, followed by incubation for 21 h. To each well, 5 ng/mL (final concentration) lipopolysaccharide (LPS, Sigma-Aldrich) was added, and the cells were subsequently incubated for additional 3 h. After 24 h, the cells were lysed with 350 μL NucleoSpin cell lysis buffer (Macherey-Nagel, Düren, Germany), and total RNA was isolated and purified using the NucleoSpin RNA Plus kit (Macherey-Nagel). Total RNA was checked for purity and quantified by UV-Vis spectroscopy (Nanodrop 2000, ThermoFisher Scientific). Purified RNA was reverse transcribed using the iScript cDNA synthesis kit (Bio-Rad Laboratories, Hercules, CA, USA). The real-time polymerase chain reaction (PCR) or quantitative PCR (qPCR) was performed in duplicate for the reference housekeeping genes [β-actin (ACTB) and β-glucuronidase (GUSB)] and in triplicate for the TNF-α gene using a LightCycler® 480 (Roche, Basel, Switzerland) and the SYBR I Green® Master Mix (Roche) as reported previously (Jensen et al., 2012) with slight modifications. The concentrations of the primers for ACTB, GUSB, and TNF-α in the reaction mixture were 1.0, 0.5, and 1.0 μM, respectively. The LightCycler® 480 software v.1.5.0 (Roche) was used for crossing point (*CP*) analysis, followed by quantification relative to LPS-treated cells using the comparative ΔΔ*CP* method (Pfaffl, 2001).

### Cell Viability

RAW 264.7 cells were seeded in a 96-well plate at a density of 5,000 cells/well in a final volume of 180 μL and allowed to adhere by incubation for 4 h. Subsequently, the cells were incubated for 24 h with 20 μL test formulations in quadruplicates at eight different TNF-α siRNA concentrations (1.38–222.8 nM). The final volume in the well was 200 μL. At 24 h, the cell culture medium was withdrawn from each well. Adherent cells were washed with PBS, followed by the addition of 180 μL cell culture medium and 20 μL freshly prepared 12 mM methylthiazolyldiphenyl-tetrazolium bromide (MTT) (Sigma Aldrich). The plates were incubated for 2 h at 37°C in 5% CO_2_-95% atmospheric air. At 26 h, the cell culture medium containing MTT was removed from the wells, and the plate was thoroughly air-dried overnight in the dark before adding 200 μL dimethyl sulfoxide to solubilize formazan MTT crystals. The cell viability was determined based on the amount of formazan crystals formed in cells incubated with the test formulations vs. the control cells by measuring the absorbance at 570 nm using a microplate reader (FLUOstar OPTIMA, BMG Labtech, Germany).

### Statistical Analysis

Data were analyzed using GraphPad Prism (GraphPad Software version 8, La Jolla, CA, USA) and represented as mean values ± standard deviation (SD). Statistically significant differences were assessed by one-way analysis of variance (ANOVA) followed by a Tukey's *post-hoc* multiple comparison test. A *p*-value ≤ 0.05 was considered statistically significant.

## Results

### Identification of QTPP and CQAs

The QTPP of the TNF-α siRNA-loaded LPNs was defined (Table 2) to ensure that the desired product properties were engineered into the nanoparticles already during the design phase (Colombo et al., 2018). Hence, the QTPP served as a guide for the optimization of formulation and process parameters to ensure that CQAs were within the desired range. The selected CQAs define the criteria of the liquid formulation encompassing safety, efficacy, and quality. The following CQAs were identified: (i) *z*-average, (ii) PDI, (iii) zeta potential, (iv) encapsulation efficiency, (v) loading of siRNA, (vi) *in vitro* transfection efficiency, and (vii) *in vitro* cell viability.

**Table 2:** Quality target product profile (QTPP) of TNF-α siRNA-loaded lipidoid-polymer hybrid nanoparticles (LPNs).

**Response**	**Target**	**Explanation**
*z*-average	<200 nm	Maximal uptake by macrophages
PDI	<0.200	Predictable particle behavior
Zeta potential	>0.0 mV	Enhanced interaction with macrophages
Encapsulation efficiency	>60.0%	Pharmacoeconomic consideration
Loading efficiency	>6.0 μg/mg LPNs	Reduced effective dose of siRNA and delivery system
Gene silencing efficiency (IC_50_)	<20.0 nM	
Cell viability	>200 nM	

### Effect of Independent Variables on the Physicochemical Properties of LPNs

To investigate the effect of one of the independent variables, i.e., the L_5_ content (15, 20, and 25%), on the physicochemical properties of the LPNs, the other variable, i.e., the L_5_:TNF-α siRNA ratio, was kept constant at four different levels (5.0:1, 7.5:1, 10.0:1, and 15.0:1) (Table 3). Significant differences were not observed in the *z*-average and PDI, and the values were within the range of the QTPP. A ~1.9-fold increase in the loading was observed when the L_5_ content was increased from 15 to 25% at L_5_:TNF-α siRNA ratios from 7.5:1 to 15.0:1. However, the loading dropped to 1.5-fold when the ratio was 5.0:1. As expected, the zeta potential increased with the L_5_:TNF-α siRNA ratio due to an increase in the content of cationic L_5_.

**Table 3:** Physicochemical properties of the formulations used for the I-optimal design. Data are shown as mean values ± SD of three independent formulation batches and three technical replicates (*N* = 3 and *n* = 3).

**L_**5**_ content (%, w/w)**	**L_**5**_:TNF-α siRNA weight ratio**	***z*-average (nm)**	**PDI**	**Zeta potential (mV)**	**Encapsulation efficiency (%)**	**TNF-α siRNA loading (μg siRNA/mg LPNs)**	**Fold reduction in IC_**50**_ value for TNF-α gene silencing^∧^**
15	5.0:1	202.5 ± 12.6^#^	0.126 ± 0.038^#^	6.7 ± 0.6^*^	71.6 ± 8.6^#^	23.7 ± 0.6^***^	3.4
	7.5:1	195.0 ± 1.5	0.102 ± 0.014	13.8 ± 2.0	64.4 ± 13.0	12.9 ± 2.6^**^	1.7
	10.0:1	194.6 ± 1.9	0.100 ± 0.027	13.7 ± 3.0	67.2 ± 2.8	10.1 ± 0.4	3.3
	15.0:1	194.2 ± 3.2	0.113 ± 0.024	19.6 ± 1.7	67.1 ± 4.3	6.7 ± 0.4	4.5 ± 1.2^#^
20	5.0:1	202.0 ± 11.4	0.119 ± 0.010	19.5 ± 1.8	73.5 ± 3.6	29.4 ± 1.4^***^	3.1
	7.5:1	188.2 ± 4.4	0.106 ± 0.032	22.2 ± 8.6	73.8 ± 9.4	19.7 ± 2.5^***^	1.6
	10.0:1	189.7 ± 2.9	0.116 ± 0.028	26.5 ± 2.7	76.7 ± 9.4	15.3 ± 1.8^***^	2.6
	15.0:1	190.7 ± 1.9	0.125 ± 0.038	30.9 ± 1.0^*^	78.0 ± 5.0	10.4 ± 0.7	3.22 ± 0.6^¤^
25	5.0:1	186.0 ± 9.8	0.118 ± 0.009	21.5 ± 5.0	71.8 ± 2.8	35.9 ± 1.4^***^	3.3
	7.5:1	187.9 ± 12.0	0.175 ± 0.082	32.0 ± 4.8^*^	73.8 ± 9.6	24.6 ± 3.2^***^	1.3
	10.0:1	181.2 ± 7.3	0.148 ± 0.051	33.3 ± 7.2^**^	74.3 ± 9.5	18.6 ± 2.4^***^	1.9
	15.0:1	181.0 ± 16.8	0.166 ± 0.009	39.0 ± 5.7^***^	74.3 ± 3.0	12.4 ± 0.5^**^	4.6

∧ *Reduction relative to non-encapsulated TNF-α siRNA, where the IC_50_ value for silencing TNF-α expression for non-encapsulated TNF-α is 63.7 nM*.

# *N = 3, ¤N = 4*.

* *p < 0.05*,

** *p < 0.01*,

*** *p < 0.001 compared to 15%, 15.0:1*.

The physicochemical properties of the formulations were explored further by keeping the L_5_ content constant and varying the L_5_:TNF-α siRNA ratio. The *z*-average and PDI of the LPNs remained almost constant at all tested L_5_:TNF-α siRNA ratios (Table 3). In contrast, a significant decrease in the zeta potential was observed as the L_5_:TNF-α siRNA ratio was decreased (corresponding to a higher amount of siRNA without increasing the L_5_ content). Hence, the L_5_ content and the L_5_:TNF-α siRNA ratio influence the zeta potential and the siRNA loading. The encapsulation efficiency was neither affected by the L_5_ content nor the L_5_:TNF-α siRNA ratio, and no significant differences were observed. However, the responses were within the QTPP (>60%). Based on these results, the L_5_ content relative to the final batch weight (%, w/w, 15, 20, and 25%) and the L_5_:TNF-α siRNA ratio (5.0:1, 7.5:1, 10.0:1, and 15.0:1) were selected for inclusion in a systematic DoE.

### Effect of TNF-α siRNA-Loaded LPNs on TNF-α mRNA Expression

**Figure 1:**
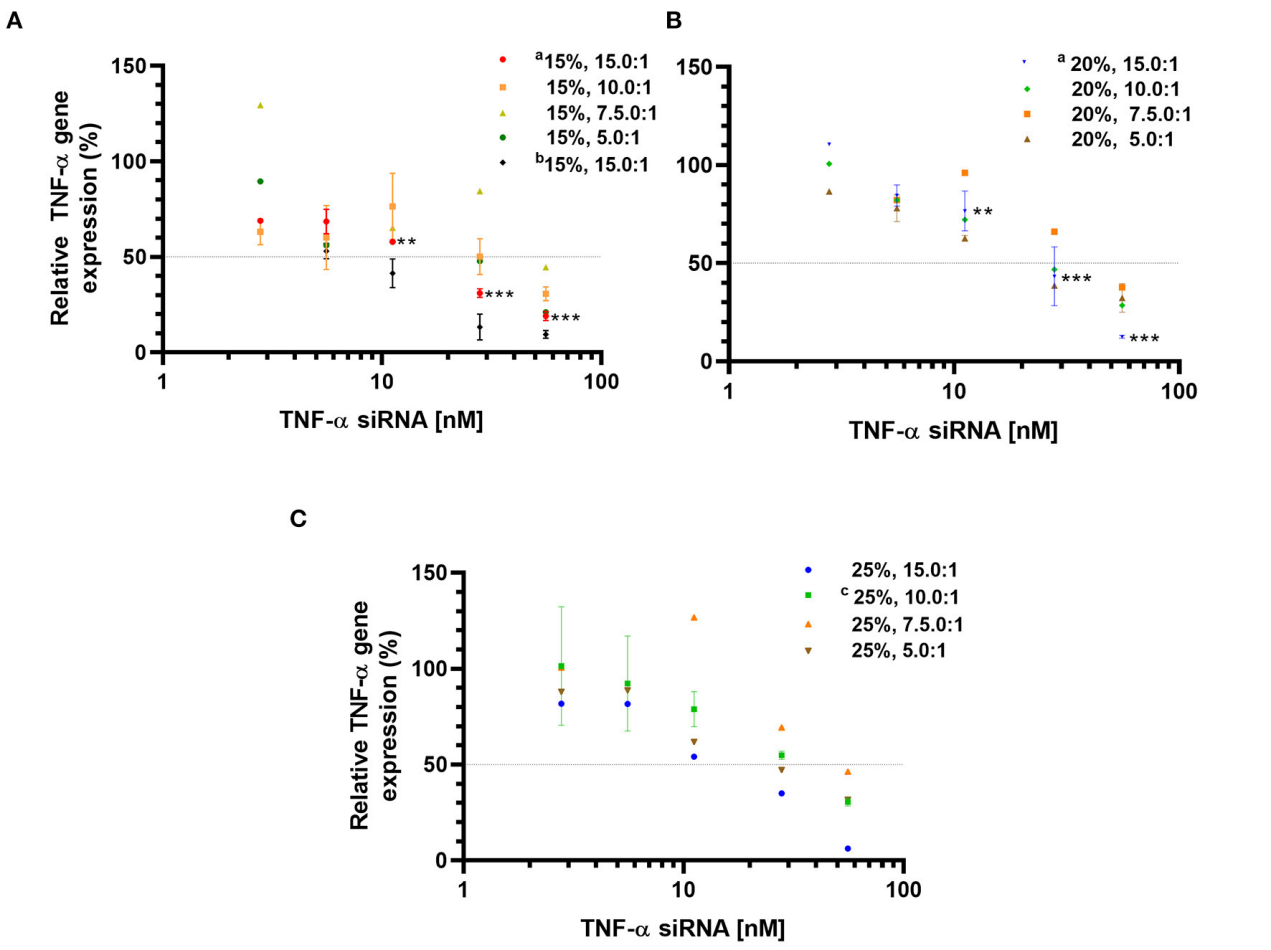
Relative TNF-α mRNA expression determined by qPCR in LPS-activated RAW 264.7 macrophages incubated with TNF-α siRNA loaded formulations. Formulations with an L_5_ content of 15% **(A)**; 20% **(B)**, and 25% **(C)**. Data is normalized to non-transfected, LPS-treated cells (100% TNF-α mRNA expression). Data is represented as mean values ± SD (^a^*N* = 3, *n* = 3, ^c^*N* = 2, *n* = 3, 5 ng LPS/mL; ^b^*N* = 3, *n* = 3, 1.25 ng LPS/mL and unmarked *N* = 1, *n* = 3), where *N* = independent formulations with duplicates of each concentration during transfection, *n* = technical replicates. **p < 0.01, ***p < 0.001 compared to 5.58 nM TNF-α siRNA concentration in LPNs.

The TNF-α gene silencing activity was evaluated at the mRNA level in the murine macrophage cell line RAW 264.7 activated with LPS. The TNF-α siRNA-loaded LPNs induced concentration-dependent TNF-α gene silencing (Figure 1). However, the magnitude of the silencing effect was specific for each formulation. Overall, both the L_5_ content and the L_5_:TNF-α siRNA ratio affected the gene silencing. At an siRNA concentration of 5.6 nM for LPNs having 15% L_5_ content, the TNF-α mRNA expression was ~60%. In contrast, the expression was at least 80% with the remaining LPNs. At the highest tested siRNA concentration (55.8 nM), the TNF-α mRNA expression generally decreased as the L_5_ content increased (Figure 1) at a constant L_5_:TNF-α siRNA ratio. At a constant L_5_ level, as the amount of siRNA increased in the LPNs, the TNF-α mRNA expression decreased from 15.0:1 to 7.5:1. Interestingly, cells incubated with LPNs having a ratio of 5.0:1 had lower TNF-α mRNA expressed for reasons that need to be investigated. As expected, a decrease in the LPS concentration used for activation of the macrophages from 5 to 1.25 ng/mL resulted in only 50% TNF-α mRNA expression when treated with LPNs composed of 15% L_5_ and having an L_5_:TNF-α siRNA ratio of 15.0:1 at 5.6 nM (Figure 1A). Even though the cells incubated with LPNs having an L_5_:TNF-α siRNA ratio of 7.5:1 expressed higher TNF-α gene levels compared to cells incubated with other LPNs, it was interesting to note that at an siRNA concentration of 55.6 nM, the TNF-α mRNA expression was ~44.6 (Figure 1A), 37.7 (Figure 1B), and 46.0% (Figure 1C), hence displaying a sharp increase in the silencing effect relative to lower concentrations. The TNF-α mRNA levels were reduced by 93.8% in cells incubated with the formulation containing 25% L_5_ and an L_5_:TNF-α siRNA ratio of 15.0:1 at the highest tested TNF-α siRNA concentration (Figure 1C). It appears that as the L_5_ content increases, the TNF-α mRNA expression increases at the lower siRNA concentrations tested (Figure 1). Overall, LPNs containing an L_5_ content of 15, 20, and 25% with L_5_:TNF-α siRNA ratio of 15:1 were most efficient in causing a significant concentration-dependent decrease in TNF-α mRNA expression.

### Effect of TNF-α siRNA-Loaded LPNs on Cell Viability

**Figure 2:**
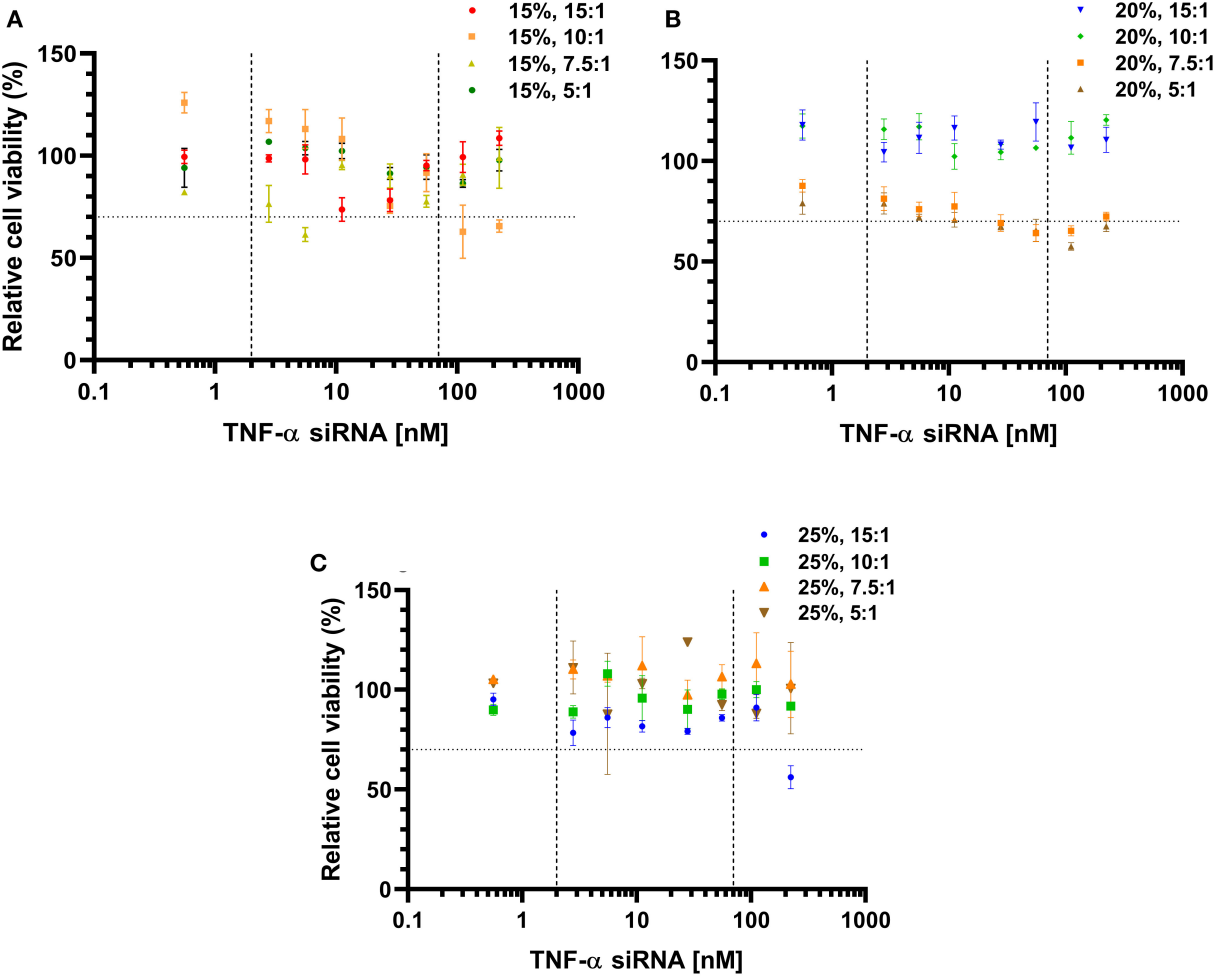
Cell viability of RAW 264.7 macrophages incubated with TNF-α siRNA-loaded lipidoid-polymer hybrid nanoparticles (LPNs). Formulations with an L_5_ content of 15% **(A)**; 20% **(B)**, and 25% **(C)**. Data was normalized to un-treated cells. The dotted horizontal line indicates the cytotoxic potential according to ISO guidelines (Srivastava et al., 2018), while the vertical dashed lines indicate the concentrations of the formulations tested for gene silencing as well. Data is represented as the mean of one independent batch tested in quadruplicates per test concentration.

To investigate whether incubation with TNF-α siRNA-loaded LPNs influenced the viability of RAW 264.7 macrophages and hence affect the measured TNF-α gene silencing effect, the viability was quantified using the MTT assay. The cell viability appeared to drop between 5.6 and 55.8 nM siRNA concentrations for all formulations with an L_5_ content of 15% (Figure 2). However, for the formulations displaying an L_5_:TNF-α siRNA wt. ratio of 10.0:1, the viability dropped at 27.8 nM and continued to decrease below 70% after a brief increase at 55.8 nM (Figure 2A). Formulations having an L_5_ content of 20% and L_5_:TNF-α siRNA ratios of 15.0:1 and 10.0:1 exhibited no cytotoxicity, evident by almost 100% cell viability at all tested concentrations. Between TNF-α siRNA concentrations of 11.2 and 222.8 nM, the cell viability was ~70% and appeared to increase from 111.4 nM (Figure 2B). Formulations having an L_5_ content of 25% did not appear to be cytotoxic based on the nearly constant cell viability >80% at all tested siRNA concentrations. However, for the formulation having a wt. ratio of 15.0:1, the viability was above 75% up to 111.4 nM siRNA concentration but then the viability steeply declined to around 56% (Figure 2C). Of all the LPNs tested for cell viability, only a few of them (15%, 10.0:1 and 7.5:1; 20%, 7.5:1, and 5.0:1; 25% 15.0:1) seemed to have cytotoxic potential (Figure 2) but none of them caused >50% loss in cell viability even at 222.8 nM. Thus, in terms of cytotoxicity and TNF-α gene silencing effect, formulations having an L_5_ content and L_5_:TNF-α siRNA wt. ratio of 15%, 15.0:1 and 20%, 15.0:1 exhibited a balanced profile and were consequently used for model validation.

### Contour Profiling of Response Variables

**Figure 3:**
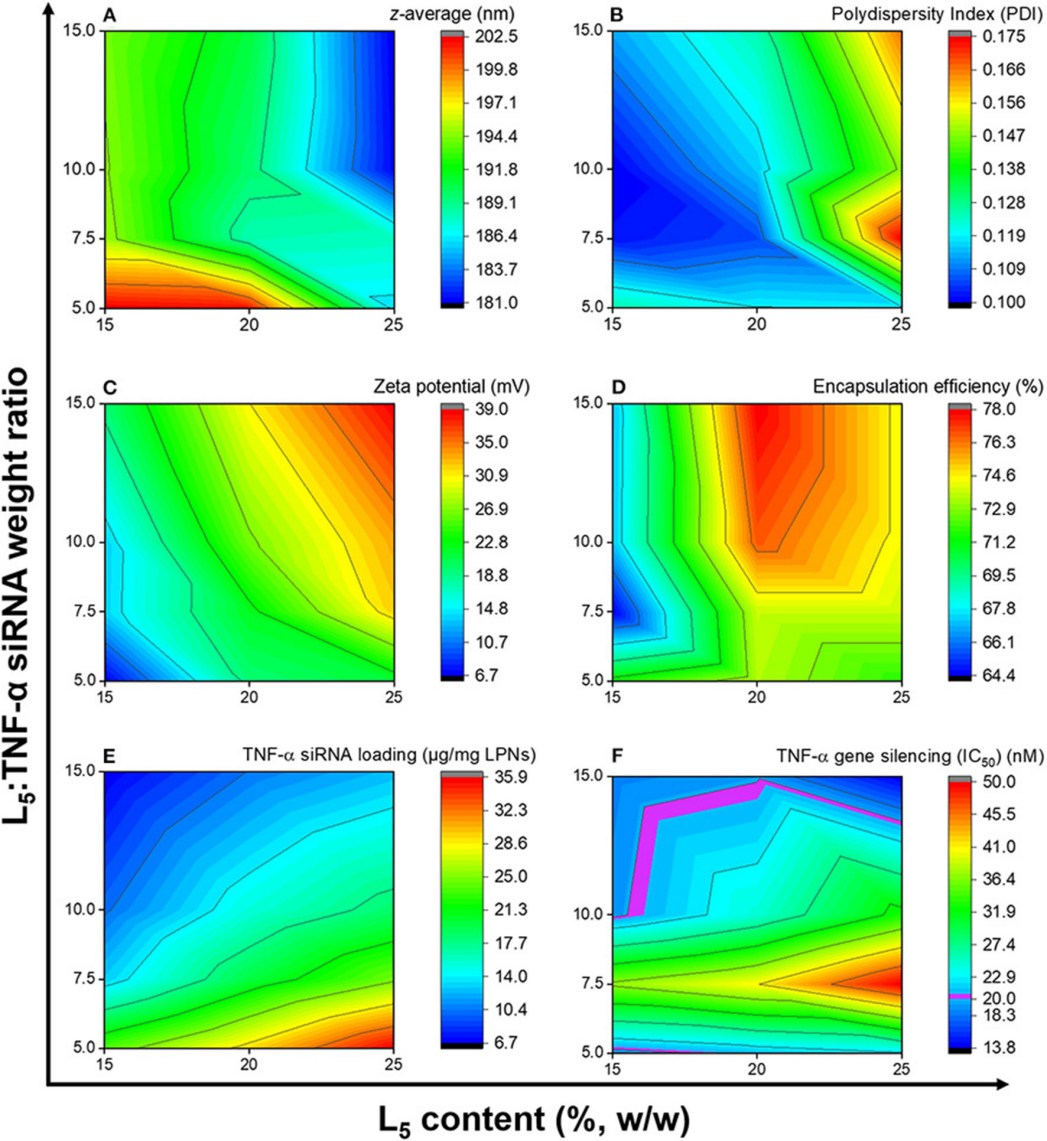
Contour plots showing the effects of the independent variables on the *z*-average **(A)**, polydispersity index **(B)**, zeta potential **(C)**, encapsulation efficiency **(D)**, TNF-α siRNA loading **(E)**, and TNF-α gene silencing **(F)**. Data is based on the mean of three independent formulations (*N* = 3, *n* = 3) for **(A–E)**, while for **(F)** it is based on mean of duplicates of transfection well and triplicates of qPCR reaction for one formulation.

Formulations from the design space (Table 1) were prepared, and responses to the independent variables were assessed and compared to the QTPP (Table 2). Contour plots were constructed for each of the response variables as a function of the L_5_ content (%w/w) and the L_5_:TNF-α siRNA ratio (Figure 3). The differences in *z*-average were not significant in the design space (Figure 3A). This suggests that the *z*-average is not affected by the independent variables but may rather be dependent on the process parameters for preparing the particles. However, the *z*-average decreased considerably when the L_5_ content was higher than 20% and the L_5_:TNF-α siRNA ratio was >7.5:1, and vice versa. There was a gradual increase in the PDI (from ~0.100 to 0.175) as the L_5_ content increased from 15 to 25% (Figure 3B). A minor increase in the PDI was observed when the L_5_:TNF-α siRNA ratio decreased at an L_5_ content of 15%. The zeta potential increased gradually from 6.7 mV to a maximum of 39.0 mV (Figure 3C). The encapsulation efficiency remained almost constant (64–78%) throughout the design space (Figure 3D), which may indicate that the encapsulation efficiency is more dependent on the process of preparing the LPNs rather than on the independent variables. The siRNA loading was affected by both independent variables (Figure 3E). The L_5_:TNF-α siRNA ratio displayed a greater impact on the loading, which was expected as a higher amount of siRNA would correspond to increased loading per weight of LPNs. To test the performance of the formulations loaded with TNF-α siRNA in a biologically relevant system, the IC_50_ values were calculated as a function of the TNF-α siRNA concentration in formulations responsible for half maximal inhibition of TNF-α mRNA expression in macrophages (Figure 3F). The IC_50_ values for transfection efficiency of all formulations ranged from 10.2 to 50.0 nM. Formulations displaying the highest L_5_:TNF-α siRNA wt. ratio, i.e., least amount of TNF-α siRNA relative to L_5_, at 15, 20, and 25% L_5_ had transfection efficiencies below 20 nM. The IC_50_ value for naked TNF-α siRNA was found to be 63.7 nM. Fold-change in TNF-α mRNA inhibition relative to naked siRNA was obtained by the ratio of IC_50_ values of TNF-α siRNA-loaded LPNs and naked TNF-α siRNA. This corresponds to an siRNA dose-reduction in obtaining similar responses. At a constant L_5_ content, the fold-change increased as per the quadratic model fitting (Tables 3, 4) from 7.5:1 to 15.0:1 ratio. When the L_5_:TNF-α ratio was kept constant, the fold-change generally increased with an increase in the L_5_ content. Interestingly, LPNs with an L_5_ content of 15 and 25% had similar IC_50_ values.

**Table 4:** Model fitting parameters for significant responses^*^.

**Parameter**	**Zeta potential**	**siRNA loading**	**Gene silencing (IC_**50**_)**
*p*-value	<0.0001	<0.0001	0.0013
*R*^2^	0.739	0.940	0.938
Adjusted *R*^2^	0.719	0.922	0.893
Predicted *R*^2^	0.657	0.900	0.768
Adequate precision	12.8	23.4	13.0
Model	2FI	Quadratic	Quadratic

* *Formulations displaying an L_5_:TNF-α siRNA wt. ratio of 5.0:1 were excluded from the design*.

### Mathematical Modeling of Responses

The obtained responses (Supplementary Table 3) were fitted with an appropriate, non-aliased model using ANOVA. Although the model was significant for the *z*-average and the PDI (*p* = 0.0042 and 0.0177), the *R*^2^ was only −0.3 and 0.27, respectively, hence these responses were excluded from the analysis. For all modeled responses (Table 4), the adjusted *R*^2^ was in reasonable agreement with the predicted *R*^2^ (the difference was <0.2), while the adequate precision was higher than four. In addition, the lack of fit was not significant for all modeled responses. Formulations displaying an L_5_:TNF-α siRNA wt. ratio of 5.0:1 were excluded from the mathematical modeling because the IC_50_ values for gene silencing fit the model equations linearly from 7.5:1 to 15.0:1 ratio.

### Identification of the Optimal Operating Space

**Figure 4:**
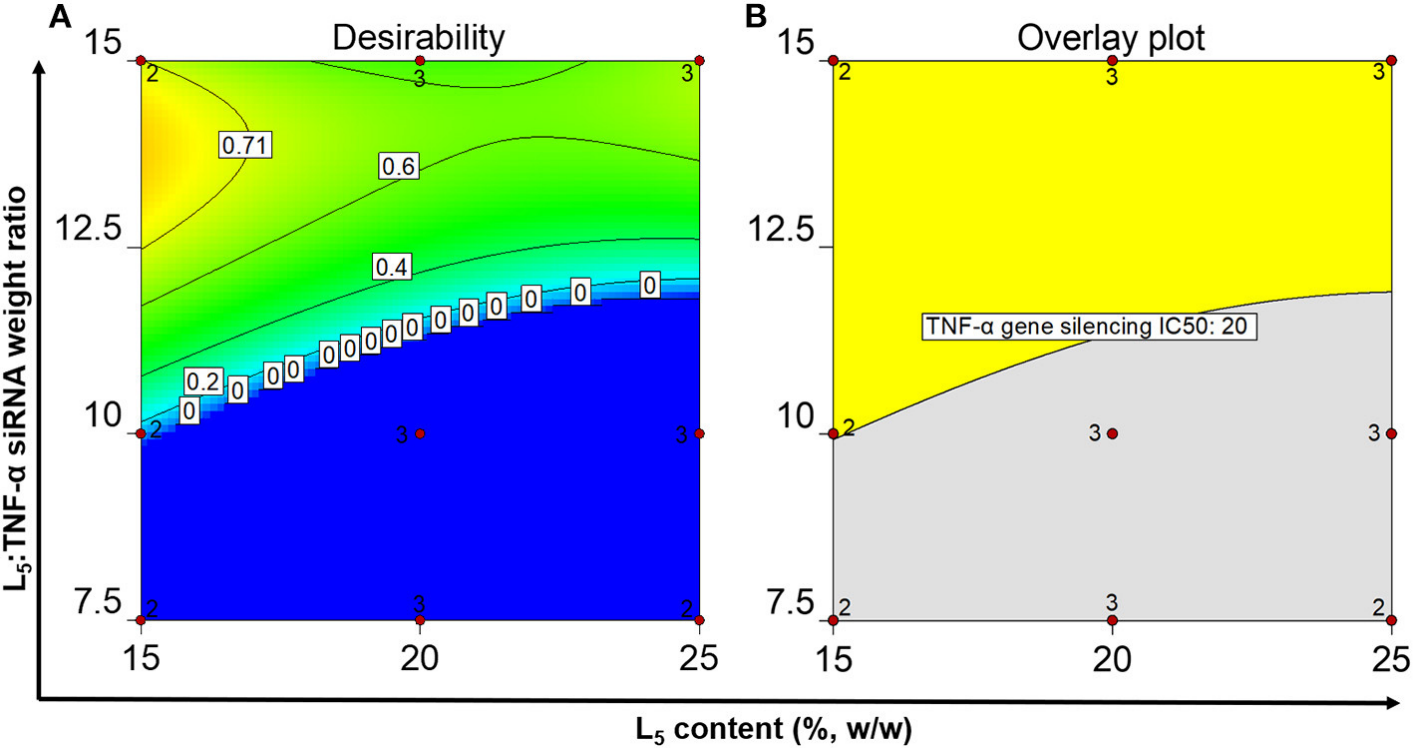
Numerical optimization plot showing the desirability of the identified formulations in the optimal operating space **(A)** and graphical optimization plot displaying the optimal operating space **(B)** after setting the quality criteria.

A desirability plot was constructed with parameter inputs (Supplementary Table 3) from the QTPP (except cell viability, for which the IC_50_ could not be determined due to negligible effects if the formulations on cell viability) and the prediction model analyzed using ANOVA (Figure 4). The region shaded toward dark yellow (Figure 4A) constitutes the optimal L_5_ content and L_5_:TNF-α siRNA weight ratio where all the responses show the most optimal values. The edge of failure is the boundary between the dark blue region and the other colored regions, where either one or several responses do not fulfill the set criteria. The light blue shaded region (Figure 4A) indicates that all criteria defined in the QTPP were met but were less optimal relative to the green and yellow region. Ten formulations displaying a desirability score ranging from 0.671 to 0.796 were identified (Supplementary Table 4). An overlay plot (Figure 4B) was constructed, which shows all possible combinations of L_5_ content and L_5_:TNF-α siRNA ratio (Table 4). In other words, this is the OOS, and a formulation selected from this region is most likely to have the desired characteristics.

### Validation of Responses

Although the desirability scores (Figure 4A) show that the point formulations with the most desirable properties are 15%, 13.5:1 and 25%, 15.0:1, they do not account for the cell viability since the IC_50_ values could not be calculated due to lack of typical log dose-response relationship at the tested concentrations. To exclude potential cytotoxic formulations at the tested concentrations (Figure 2), two formulations having L_5_ contents and L_5_:TNF-α siRNA wt. ratios of 15 and 20%, 15.0:1 were formulated in triplicates to validate the OOS. The physicochemical properties of the formulations and the IC_50_ values for TNF-α gene silencing were determined and compared to the point formulations from numerical optimization using specific criteria (Tables 4, 5). Using one-way ANOVA, no statistically significant differences were observed between the *z*-average, PDI, and TNF-α gene silencing for these two formulations (Table 5). However, there was a significant difference between the zeta potential (*p* < 0.05). The IC_50_ values of TNF-α siRNA-loaded LPNs with L_5_ content of 15 and 20% having an L_5_:TNF-α siRNA ratio of 15.0:1 relative to non-encapsulated siRNA were 4.5 ± 1.2 and 3.2 ± 0.6-fold times lower, respectively (Table 5).

**Table 5:** Validation of two, point formulations from the optimal operating space.

**L_**5**_ content (%, w/w)**	**L_**5**_: TNF-α wt. ratio**	***z*-average (nm)**	**PDI**	**Zeta potential (mV)**	**Encapsulation efficiency (%)**	**TNF-α siRNA loading (μg/mg LPNs)**	**TNF-α gene silencing IC_**50**_ (nM)**	**Desirability**	**Fold reduction in IC_**50**_ value for TNF-α gene silencing**
15.0	15.0:1	194.7 ± 3.3	0.113 ± 0.024	19.6 ± 1.7	67.1 ± 4.3	6.7 ± 0.4	15.1 ± 4.1	0.712	4.5 ± 1.2
20.0	15.0:1	190.7 ± 1.9	0.125 ± 0.038	30.9 ± 1.0	78.1 ± 5.1	10.4 ± 0.7	20.9 ± 4.6	0.569	3.2 ± 0.6

*Data are represented as mean values ± SD of independent formulations (N = 3-4)*.

## Discussion

Adopting a QbD approach ensures the quality of medicines by employing statistical, analytical, and risk management methodologies in the design, development, and manufacturing of medicines (Lawrence et al., 2014). Such an approach helps in identifying all potential sources of variability that may affect a process and/or a formulation and aids in meeting the predefined characteristics from the very beginning. Thus, applying these principles when designing a formulation of TNF-α siRNA-loaded LPNs may ensure a high-quality and robust formulation displaying high siRNA loading, optimal physicochemical properties, high gene silencing efficiency, and low toxicity. Hence, the specific purpose of this study was to optimize the loading of TNF-α siRNA in LPNs using a QbD approach, where the L_5_ content relative to the PLGA content in the formulation was varied, along with the L_5_:TNF-α siRNA weight ratio, by keeping the total weight of the solids (L_5_ + PLGA) constant. This was followed by assessment of all formulations for their effect on TNF-α gene silencing and cell viability in activated RAW 264.7 macrophages.

The physicochemical properties of the LPNs were dependent on the starting amounts of lipid and siRNA. First, the effect of the L_5_ content on various responses was identified by keeping the L_5_:TNF-α siRNA ratio constant. Based on these one-factor-at-a-time experiments, it was established that the L_5_ content significantly affects the zeta potential and, in some cases, the *z*-average and PDI. The *z*-average for LPNs formulated using the optimized DESE method used in this study is expected to be ~200 nm. To confirm the effect on *z*-average and PDI at higher L_5_ content (30 and 40%) by keeping the L_5_:TNF-α siRNA ratio 15.0:1, two formulations were prepared, and it was found that the *z*-averages were 159.1 and 154.8 nm with PDIs of 0.216 and 0.272, respectively (Supplementary Table 2). These differences were statistically significant (*p* < 0.01). One explanation could be that as the amount of L_5_ is increased, the PLGA content decreases, which means a lower concentration of the latter in the organic phase during the preparation process and consequently a reduction in particle size. This may be attributed to a decrease in solvent viscosity, increase in diffusion coefficient, and the decrease in PLGA content in the droplets of the organic phase resulting in smaller particles after solvent evaporation (Feczkó et al., 2011). The PLGA concentration in the organic phase is shown to influence particle size. An increase in the lipid content could lead to a heterogeneous population of lipoplexes and LPNs, thus increasing the PDI. This can be explained by the fact that at a lipid:polymer weight ratio of 10:90, the amount of lipid is within a range to cover the entire surface of the hydrophobic PLGA core. Similar observations have been reported previously, where the excess lipids have been speculated to increase above the critical micellar concentration, resulting in the assembly of liposomes, hence suggesting the coexistence of liposomes and hybrid nanoparticles (Zhang et al., 2008).

The encapsulation efficiency remained in the range of 60-80%, which is similar to the encapsulation efficiency of enhanced green fluorescent protein (eGFP) siRNA-loaded LPNs as we have reported previously (Thanki et al., 2017). However, the inclusion of L_5_ in the nanoparticles significantly increased the encapsulation efficiency compared to the encapsulation efficiency in non-modified PLGA nanoparticles (data not shown), in agreement with previous reports (Thanki et al., 2017). PLGA is a hydrophobic polymer composed of lactic and glycolic acid monomers with pK_a_ values of 3.86 and 3.83, respectively (Yoo and Mitragotri, 2010). The pH value of the water phase during LPN preparation is ~6.5. Hence, at this pH, the carboxylic acid end groups of PLGA exist in a deprotonated state. These anionic residues are not favorable for interaction with the negatively charged phosphate backbone of siRNA. Differences between *z*-average and encapsulation efficiencies were not significant and were within the desirable limits.

An increase in the L_5_ content from 15 to 25% resulted in ~2 to 4-fold increase in the zeta potential at all tested L_5_:TNF-α siRNA ratios (Table 3). The zeta potential is an indirect measure of the surface charge of the nanoparticles. It is one of the physicochemical properties that influences particle uptake by macrophages (Alexis et al., 2008). In one study, it was found that both positive and negative surface charges were promoting uptake of carboxymethyl hydrochloride-grafted polymeric nanoparticles (He et al., 2010). These results suggest that surface charge-dependent uptake is highly variable and depends on the type of formulation, uptake mechanisms, and the biological system. Since the LPNs are composed of cationic lipids, it is imperative to know the zeta potential as it can have a possible effect on the uptake of particles and for this reason, it was set to >0 mV in the QTPP (Table 2). In addition, the zeta potential is also a measure of particle colloidal stability, where higher magnitude suggests enhanced colloidal electrostatic stability (Shah et al., 2015). Furthermore, the intended site of action of these LPNs is the alveoli, which houses the alveolar macrophages (Byrne et al., 2015). These LPNs have been engineered as spray-dried particles intended for inhalable alveolar delivery to silence TNF-α gene in macrophages (Dormenval et al., 2019), thus circumventing the barriers of systemic delivery related to excess surface charge. Overall, the results are in agreement with previous reports where the zeta potential increased linearly with an increase in the molar composition of cationic lipids (Smith et al., 2017).

When keeping the L_5_ content constant and varying the L_5_:TNF-α siRNA ratio, it was observed that the siRNA loading decreased 3-fold when the L_5_:TNF-α siRNA ratio was increased from 5.0:1 to 15.0:1. The L_5_:TNF-α siRNA ratio displayed a greater impact on the siRNA loading, which was expected as a higher initial amount of siRNA would correspond to an increased loading per weight of the LPNs. The encapsulation efficiency increased (from 64.4 to ~80%) as the L_5_ content increased from 15 to 25%, highlighting the role of L_5_ in electrostatic interactions with siRNA. The relatively lower encapsulation efficiency could be due to the high pH (7.5) during the emulsification process as L_5_ might not be fully protonated (de Groot et al., 2018). In addition to the amount of cationic lipid, the overall performance of the system is also dependent on the dose of siRNA that reaches the cytosol of the cell. Thus, siRNA loading is a response worth investigating. The loading was expected to increase because even at the same weight ratio and a higher L_5_ content, the absolute amount of siRNA was higher. Thus, to have a direct comparison, formulations with L_5_ contents of 15 and 20% with L_5_:TNF-α siRNA ratios of 7.5:1 and 10.0:1, respectively, contained equal amounts of TNF-α siRNA (300 μg). No statistically significant differences were observed with respect to TNF-α siRNA loading between the two formulations. Similar results were obtained for LPNs loaded with an antisense oligonucleotide (ASO) (Thanki et al., 2019c). The encapsulation efficiency is important in terms of performance and pharmacoeconomics. Hence, a value higher than 60% was set for the encapsulation efficiency, while a higher siRNA loading (>6 μg siRNA/mg LPNs) would have a positive impact on the gene silencing effect and reduction in cytotoxicity. The increase in loading without compromising the particle properties suggests that the electrostatic charge condensation between the siRNA and the amine headgroups of L_5_ is not complete and that more siRNA could be complexed. However, in general, excess siRNA may not necessarily translate into higher gene silencing because the cationic character of the lipid decreases, eventually resulting in poor transport across the cell membrane and thus poor transfection (Fröhlich, 2012). Based on these observations, it is evident that both the L_5_ content and the L_5_:TNF-α siRNA ratio affect the physicochemical properties of the LPNs, hence they were included in the systematic QbD-based optimization of the LPNs.

Nucleic acid delivery has traditionally been hindered by the toxicity associated with their delivery systems. In the past years, novel carrier systems and analogs have been developed to overcome this problem (Love et al., 2010). *In vitro* studies using the MTT assay exhibit that the LPNs, in general, do not have a cytotoxic potential at the measured concentration ranges. The toxicity is predominantly induced by the positively charged lipid component of the nanoparticulate carrier. However, the use of synthetic lipids or lipidoids help overcome these delivery challenges (Akinc et al., 2008). No differences in cell viability were found (except at 11.2 nM) between the LPNs having the highest (5.0:1 wt. ratio, 6.5 ± 0.6 mV) and least amount of siRNA (15.0:1 wt. ratio, 20.4 ± 1.5 mV) highlighting that the zeta potential of a formulation measured in the dispersion medium is not the only determinant of the toxicity. Dose response of siRNA LPNs demonstrated lack of cytotoxic potential for different formulations tested at certain concentrations for gene silencing (Figures 2A–C), and only a few of them (15%, 10.0:1 and 7.5:1; 20%, 7.5:1; 25% 15.0:1) seemed to have cytotoxic potential. The possibility of potential apoptotic and necrotic effects of the particles should not be disregarded since it may not be apparent from the MTT assay. However, at TNF-α siRNA concentrations of 100 and 200 nM, no significant apoptotic or necrotic cells were observed for 15%, 15.0:1 LPNs (Jansen et al., 2019).

In the present study, we identified the effect of the independent variables on the cell viability where all the tested formulations had comparable siRNA concentrations. It was found that non-encapsulated TNF-α siRNA and scrambled siRNA did not affect the cell viability at the tested concentrations. Thus, the toxicity of the formulations is most likely caused by the L_5_ component. It has been reported that certain lipidoids. e.g., DLinDMA, at a concentration of 1 μg/mL of siRNA induce a significant increase in apoptotic macrophages (Basha et al., 2011). Cytotoxicity is highly affected by various factors, and one of them is the cell type (Nakamura et al., 2016). The concentration of TNF-α siRNA in the most optimal formulations required to knock down between 60 and 69% of TNF-α was found to be 27.8 nM, while a concentration of 55.8 nM was required for >80% knockdown. At these concentrations, none of the most optimal formulations displayed any cytotoxic potential. The TNF-α gene silencing profile was found to be quite similar to previously published results (Jansen et al., 2019).

To study the effect of the independent variables on the TNF-α mRNA expression, 12 formulations with different L_5_ content and L_5_:TNF-α siRNA ratios were tested at five concentrations of encapsulated TNF-α siRNA. All formulations, except the ones displaying an L_5_:TNF-α siRNA ratio of 7.5:1, exhibited IC_50_ values 2.6- to 4.6-fold lower than the LPNs loaded with negative control siRNA and the non-loaded TNF-α siRNA. These results show that using LPNs as a delivery system is clearly advantageous and the silencing is due to the specific sequence of TNF-α siRNA. This may be due to the fact that non-loaded TNF-α siRNA cannot cross the cell membrane (De Paula et al., 2007; Kim et al., 2010). The silencing mediated by non-encapsulated siRNA might be related to damage of the cell membranes upon harvesting, as the siRNA (and also the LPNs) are incubated with cells immediately after harvesting (Jensen et al., 2012). We found a linear increase in TNF-α gene silencing effect when the L_5_:TNF-α siRNA ratio was increased from 7.5:1 to 15.0:1 at 15, 20, and 25% L_5_ content. Interestingly, LPNs with a 5.0:1 ratio silenced TNF-α gene expression more efficiently than 7.5:1. We do not fully understand the reason for these results, which needs to be addressed in future studies. Although the IC_50_ values are quite different for every formulation, it is evident that at the highest tested concentration of siRNA, the gene expression of TNF-α in LPS-activated macrophages treated with LPNs containing 15, 20, and 25% L_5_ and having an L_5_:TNF-α siRNA ratio of 15.0:1 was only 19, 12, and 6%, respectively. Similar observations have been documented previously where increasing the lipidoid content resulted in more efficient silencing of Factor VII mRNA (Akinc et al., 2009; Basha et al., 2011). This suggests that the positive charge of the lipid is not the only determinant of transfection efficiency and thus gene silencing, and that endosomal escape can also be a greater factor governing the efficacy (Raemdonck et al., 2008). The IC_50_ values are highly variable and depend on many factors, including the composition of the formulation (Thanki et al., 2017) and the cell lines used (Nakamura et al., 2016), hence comparison seems valid when all other variables are kept constant. Based on the current observations, LPNs composed of 15, 20, and 25% L_5_ having an L_5_: TNF-α siRNA ratio of 15.0:1 were found to be most optimal for gene silencing in macrophages, as determined from their IC_50_ values (Table 3).

To date, several different lipid carriers have been studied for siRNA delivery. It is generally challenging to compare silencing and non-specific effects obtained in different studies due to highly variable experimental conditions, e.g., cell type, LPS concentration, incubation time, and presence of serum proteins. These tend to destabilize cationic nanoparticles and thus invalidate the observations, since the actual amount of siRNA that reaches the cytosol may vary (Buyens et al., 2010). In a recent study the 15%, 15.0:1 formulation was used a lead formulation to investigate critical formulation and process parameters involved in spray drying of liquid LPN dispersions into nanocomposite microparticles. The resulting spray-dried microparticles were resuspended and compared to the original liquid LPN dispersions, and no significant differences were found in their performance in terms of physicochemical properties and *in vitro* gene silencing (Dormenval et al., 2019). Also, this formulation was dosed at a TNF-α siRNA concentration of only 5.58 nM and was shown to have a remarkable gene silencing activity *in vivo* (Jansen et al., 2019). It is interesting to compare the OOS of TNF-α siRNA loaded LPNs with that of single-stranded ASO (Thanki et al., 2019c) and an siRNA directed against eGFP (Thanki et al., 2017). The *z*-average, PDI, zeta potential, siRNA loading, and encapsulation efficiencies are almost identical with the latter, which may be due to comparable nucleotide length and similar modification patterns (Supplementary Table 1), whereas the gene silencing effect depends on both the L_5_ content and the L_5_:TNF-α siRNA weight ratio as shown from the coefficients (Supplementary Table 5). Recently, we performed a detailed mechanistic evaluation of the release kinetics of fluorescently labeled siRNA from LPNs *in vitro* and *in vivo* after pulmonary administration, which demonstrated that the sustained release of siRNA from LPNs correlates well with cell uptake and *in vivo* biodistribution (Thanki et al., 2019a). It is expected that the optimal formulations identified in the current study display *in vitro* and *in vivo* release kinetics, which is highly comparable to our previously reported findings (Thanki et al., 2019a).

The OOS was constructed based on the results obtained from the above experiments and on the criteria set in the QTPP. Ten formulations displaying a desirability score from 0.671 to 0.796 were identified. The desirability values are between 0 and 1, the latter being the most desirable. However, this term may be misinterpreted because the desirability function is fully dependent on how close the upper and lower limits are set in the QTPP. The goal of optimization is how well the set conditions meet the goals and not about gaining the highest desirability (Desirability Function, 2018).

## Conclusion

In conclusion, we demonstrate the application of a systematic QbD approach for loading therapeutic TNF-α siRNA into LPNs with optimal *in vitro* gene silencing effect and safety. We show that both the L_5_ content and the L_5_:TNF-α siRNA ratio significantly affect the zeta potential, siRNA loading and TNF-α gene silencing. It was also found that the responses to the independent variables were complex, but in general there seemed to be an optimal range for loading siRNA into LPNs. QbD with statistical analysis facilitated the modeling of these responses with a high coefficient of determination, which can be useful in predicting responses based on user input of the independent variables. The LPN formulations loaded with TNF-α siRNA showed a 1.3- to 4.6-fold reduction in the effective dose for *in vitro* gene silencing as compared to the dose required for gene silencing by non-encapsulated TNF-α siRNA, highlighting the importance of a delivery system. It was also shown that the zeta potential is not the only determinant of toxicity of a formulation and that reducing the positive surface charge does not necessarily imply reduced gene silencing in every scenario. We further show that higher siRNA loading does not necessarily correlate with higher gene silencing *in vitro*. Finally, an OOS was identified based on graphical optimization, and point formulations displaying L_5_ contents of 15 and 20% and L_5_:TNF-α siRNA weight ratio of 15.0:1 were identified that satisfied all the criteria set in the QTPP and had a balanced toxicity/efficacy profile. These results demonstrate the importance of systematic formulation design for loading therapeutic TNF-α siRNA in LPNs with optimal gene silencing and safety and support our ongoing efforts for TNF-α siRNA-mediated therapeutic management of COPD in preclinical animal models.

## Data Availability Statement

The original contributions presented in the study are included in the article/Supplementary Material, further inquiries can be directed to the corresponding author.

## Author Contributions

AL, AT, KT, and CF designed the study. AL, KT, and AW performed the laboratory work. AL, AT, KT, and CF interpreted the data. AL, AT, and CF drafted the manuscript. AL, AT, AW, KT, HF, and CF provided scientific input throughout the study period and drafting of the manuscript. All authors contributed to the article and approved the submitted version.

## Conflict of Interest

The authors declare that the research was conducted in the absence of any commercial or financial relationships that could be construed as a potential conflict of interest.
