# Supplementary material for: Optimizing the Intracellular Delivery of Therapeutic Anti-inflammatory TNF-α siRNA to Activated Macrophages Using Lipidoid-Polymer Hybrid Nanoparticles
Source: Front Bioeng Biotechnol. 2021 Jan 14;8:601155. doi: 10.3389/fbioe.2020.601155 (PMC7841370; doi:10.3389/fbioe.2020.601155)
Supplement: Supplementary file 1 [file Data_Sheet_1.docx]

**Table S1:** siRNA and primer sequences and modification patterns. Upper case letters represent ribonucleotides, lower case letters represent deoxyribonucleotides, underlined capital letters represent 2'-*O*-methylribonucleotides and p is a phosphate residue.

| **Name** | **Sense sequence / Forward primer** | **Antisense sequence / Reverse primer** |
| --- | --- | --- |
| TNF-α siRNA | 5'-pGUCUCAGCCUCUUCUCAUUCCUGct-3' | 5'-AGCAGGAAUGAGAAGAGGCUGAGACAU-3' |
| Negative control siRNA | 5'-AUCGUACGUACCGUCGUAUtt-3' | 5'-AUACGACGGUACGUACGAUtt-3' |
| TNF-α | 5'-tgcctatgtctcagcctcttc-3' | 5'-ggtctgggccatagaactga-3' |
| ACTB | 5'-cagcttctttgcagctcctt-3' | 5'-cacgatggaggggaatacag-3' |
| GUSB | 5'-agttgtgtgggtgaatggga-3' | 5'-ggaagggtatgaggggtcag-3' |

**Table S2:** Physicochemical properties of lipidoid-polymer hybrid nanoparticles (LPNs) used for modeling of the responses.

|  |  | **Factor 1** | **Factor 2** | **Response 1** | **Response 2** | **Response 3** | **Response 4** | **Response 5** | **Response 6** |
| --- | --- | --- | --- | --- | --- | --- | --- | --- | --- |
| **Run** | **Space type** | **A:L_5_ content (% w/w)** | **B:L_5_: TNF-α siRNA wt. ratio** | **z-average** | **PDI** | **Zeta potential** | **Encapsulation efficiency** | **siRNA loading** | **TNF-α gene silencing (IC_50_)** |
|  |  |  |  | **nm** |  | **mV** | **%** | **µg/mg NPs** | **nM** |
| 1 | CentEdge | 15 | 7.5:1 | 196.5 | 0.104 | 12.5 | 75.8 | 13.4 | - |
| 2 | CentEdge | 15 | 7.5:1 | 193.6 | 0.116 | 16.2 | 67.1 | 15.2 | 36.95 |
| 3 | CentEdge | 15 | 10.0:1 | 192.4 | 0.076 | 17.1 | 69.1 | 10.3 | - |
| 4 | Center | 15 | 15.0:1 | 190.9 | 0.098 | 19.3 | 70.1 | 6.9 | 10.98 |
| 5 | CentEdge | 20 | 7.5:1 | 185.4 | 0.094 | 28.2 | 71.4 | 17.6 | 40.83 |
| 6 | Vertex | 20 | 7.5:1 | 185.9 | 0.082 | 26.2 | 84.2 | 19.0 | - |
| 7 | CentEdge | 20 | 7.5:1 | 193.3 | 0.143 | 12.4 | 65.9 | 22.4 | - |
| 8 | Center | 20 | 10.0:1 | 193.1 | 0.149 | 28.8 | 73.8 | 13.8 | 24.64 |
| 9 | CentEdge | 20 | 15.0:1 | 192.9 | 0.110 | 31.4 | 80.2 | 9.6 | 16.40 |
| 10 | Vertex | 25 | 7.5:1 | 200.4 | 0.119 | 37.6 | 77.2 | 25.7 | 49.97 |
| 11 | Vertex | 25 | 7.5:1 | 186.6 | 0.145 | 28.9 | 81.2 | 27.1 | - |
| 12 | Vertex | 25 | 10.0:1 | 173.9 | 0.097 | 31.4 | 81.0 | 19.6 | 32.95 |
| 13 | Vertex | 25 | 15.1:1 | 163.1 | 0.165 | 42.6 | 76.2 | 11.8 | 13.80 |
| 14 | Edge | 20 | 15.0:1 | 189.2 | 0.09 | 31.4 | 72.3 | 10.7 | 15.64 |
| 15 | Edge | 20 | 10.0:1 | 187.9 | 0.104 | 27.2 | 87.2 | 14.8 | - |
| 16 | Interior | 25 | 10.0:1 | 188.6 | 0.200 | 27.2 | 63.5 | 15.8 | - |
| 17 | Vertex | 15 | 10.0:1 | 195.8 | 0.094 | 26.0 | 68.7 | 10.4 | 19.47 |
| 18 | CentEdge | 20 | 15.0:1 | 190.1 | 0.166 | 30.8 | 81.8 | 10.9 | 23.05 |
| 19 | Vertex | 20 | 10.0:1 | 188.2 | 0.095 | 23.4 | 69.1 | 17.4 |  |
| 20 | Vertex | 15 | 15.0:1 | 197.3 | 0.100 | 21.4 | 62.2 | 7.0 | 18.21 |
| 21 | Edge | 25 | 15.0:1 | 183.7 | 0.158 | 42.2 | 75.8 | 12.6 | - |
| 22 | CentEdge | 25 | 15.0:1 | 196.3 | 0.176 | 42.5 | 70.9 | 12.7 | - |
| 23 | Edge | 25 | 10.0:1 | 181.1 | 0.147 | 27.2 | 78.5 | 20.3 | 26.86 |

**Table S3:** Input parameters and target for numerical optimization of the design.

| **Parameter** | **Target** | **Lower limit** | **Upper limit** | **Lower weight** | **Upper weight** | **Importance** |
| --- | --- | --- | --- | --- | --- | --- |
| L_5_ content (%, w/w) | In range | 15 | 25 | 1 | 1 | 3 |
| L_5_:TNF-α siRNA wt. ratio | Maximize | 7.5 | 15.0 | 1 | 1 | 3 |
| *z*-average (nm) | In range | 163.1 | 200.4 | 1 | 1 | 3 |
| PDI | Minimize | 0.076 | 0.200 | 1 | 1 | 3 |
| Zeta potential (mV) | In range | 0.0 | 42.6 | 1 | 1 | 3 |
| Encapsulation efficiency (%) | In range | 60.0 | 87.2 | 1 | 1 | 3 |
| siRNA loading (µg/mg) | In range | 6.9 | 27.1 | 1 | 1 | 3 |
| TNF-α gene silencing (IC_50,_ nM) | In range | 10.38 | 20.00 | 1 | 1 | 3 |

**Table S4:** Point solutions for the formulations from the optimal operating space.

| **L_5_ content (%, w/w)** | **L_5_:TNF-α siRNA wt. ratio** | ***z*-average (nm)** | **PDI** | **Zeta potential (mV)** | **Encapsulation efficiency (%)** | **siRNA loading (µg/mg)** | **TNF-α gene silencing IC_50_ (nM)** | **Desirability** |
| --- | --- | --- | --- | --- | --- | --- | --- | --- |
| 15.0 | 13.8 | 193.5 | 0.100 | 20.8 | 70.2 | 7.3 | 13.25 | 0.796 |
| 15.0 | 13.8 | 193.5 | 0.100 | 20.8 | 70.2 | 7.3 | 13.22 | 0.796 |
| 15.0 | 13.9 | 193.5 | 0.100 | 20.8 | 70.2 | 7.2 | 13.29 | 0.796 |
| 15.0 | 13.9 | 193.5 | 0.100 | 20.9 | 70.2 | 7.2 | 13.33 | 0.795 |
| 15.0 | 14.0 | 193.5 | 0.100 | 20.9 | 70.1 | 7.2 | 13.37 | 0.795 |
| 15.0 | 14.1 | 193.4 | 0.101 | 21.0 | 70.1 | 7.2 | 13.54 | 0.793 |
| 15.0 | 13.6 | 193.6 | 0.100 | 20.6 | 70.2 | 7.3 | 13.03 | 0.793 |
| 25.0 | 14.8 | 183.1 | 0.155 | 40.7 | 76.3 | 12.5 | 15.81 | 0.671 |
| 25.0 | 14.7 | 183.1 | 0.155 | 40.6 | 76.3 | 12.5 | 15.80 | 0.671 |
| 25.0 | 14.8 | 183.1 | 0.155 | 40.7 | 76.3 | 12.5 | 15.84 | 0.671 |

**Table S5:** Coefficients table showing the significant/non-significant effect of independent variables (A) and (B) on responses **(*p* < 0.05)**

| **Response** | **Intercept** | **A** | **B** | **AB** | **A²** | **B²** |
| --- | --- | --- | --- | --- | --- | --- |
| *z*-average | 189.3 nm | **-5.04** | -1.24 |  |  |  |
| *p*-value |  | **0.0190** | 0.5116 |  |  |  |
| PDI | 0.122 | **0.0282** | 0.0062 |  |  |  |
| *p*-value |  | **0.0016** | 0.3982 |  |  |  |
| Zeta potential | 27.1 mV | **7.85** | **4.41** |  |  |  |
| *p*-value |  | **< 0.0001** | **0.0012** |  |  |  |
| Encapsulation efficiency | 73.7 % | 3.16 | -0.60 |  |  |  |
| *p*-value |  | 0.0880 | 0.7197 |  |  |  |
| siRNA loading | 13.0 µg/mg | **4.07** | **-5.03** | **-1.56** | -0.36 | **2.19** |
| *p*-value |  | **< 0.0001** | **< 0.0001** | **0.0060** | 0.5885 | **0.0115** |
| TNF-α gene silencing (IC_50_) | 20.3 nM | **3.65** | **-13.21** | -3.38 | -1.38 | **9.99** |
| *p*-value |  | **0.0342** | **< 0.0001** | 0.0888 | 0.5638 | **0.0099** |

**TNF-α gene silencing**

**IC_50_ (nM)**


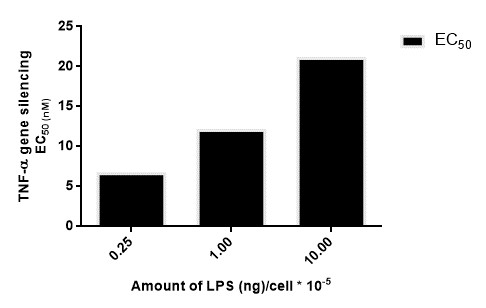


**Figure S1:** The IC_50_ value for TNF-α gene silencing in RAW 264.7 macrophages, mediated by TNF-α siRNA-loaded LPNs containing 15% L_5_ and an L_5_:TNF-α siRNA ratio of 15.0:1, increases as the LPS dose used to activate the cells is increased.
